# miR-150-5p suppresses tumor progression by targeting VEGFA in colorectal cancer

**DOI:** 10.18632/aging.101656

**Published:** 2018-11-26

**Authors:** Xiaoxiang Chen, Xueni Xu, Bei Pan, Kaixuan Zeng, Mu Xu, Xiangxiang Liu, Bangshun He, Yuqin Pan, Huiling Sun, Shukui Wang

**Affiliations:** 1 General Clinical Research Center, Nanjing First Hospital, Nanjing Medical University, Nanjing 210006, Jiangsu, China; 2 Medical College, Southeast University, Nanjing 210009, Jiangsu, China

**Keywords:** miR-150-5p, colorectal cancer, VEGFA, Akt/mTOR

## Abstract

MicroRNA-150-5p (miR-150-5p) has been implicated in tumor initiation and progression in a variety of cancers. However, its roles in colorectal cancer (CRC) remain largely unknown. In our study, a decreased miR-150-5p expression in CRC tissues was found to be associated with poor overall survival. Moreover, miR-150-5p inhibited CRC cell proliferation, migration, invasion and angiogenesis in vitro and in vivo, and its inhibitory effect could be reversed by transfection of vascular epithelial growth factor A (VEGFA) expression plasmid. Lastly, we demonstrated that miR-150-5p inactivated VEGFA/VEGFR2 and the downstream Akt/mTOR signaling pathway in CRC. Based on these results, we conclude that miR-150-5p may function as a tumor suppressor in CRC, and miR-150-5p/VEGFA axis may be a potential therapeutic target candidate in CRC treatment.

## Introduction

Colorectal cancer (CRC) is one of the most-prevalent malignancies and leading causes of cancer-related death in the world [1]. Much improvement has been made in diagnosis, treatment and understanding of the molecular mechanisms of CRC, but the prognosis for patients with CRC remains poor [2,3]. Therefore, exploration of the molecular mechanisms involved in the initiation and progression of CRC is urgently needed.

microRNAs (miRNAs), a class of conserved noncoding small RNAs, function as posttranscriptional regulators of gene expression in tumor initiation and progression by binding to the 3’-untranslated region (UTR) of target mRNA [4,5]. Recent increasing evidence has confirmed that dysregulated miRNAs play key roles in multiple biological processes in CRC [6–8], including cell proliferation, drug resistance, apoptosis, metastasis and angiogenesis and have been identified as potential diagnostic, prognostic and therapeutic biomarkers in CRC diagnosis and treatment.

miR-150-5p, a cancer-related miRNA, has been reported to be aberrantly expressed in various cancers [9,10]. Zhang Z, et al. [11] have reported that miR-150 is increased in cervical cancer and promotes cell proliferation, migration and invasion by targeting PDCD4. Zhang L, et al. [12] have reported that miR-150 is upregulated in non-small cell lung cancer (NSCLC) cells and significantly associated with survival, and increased expression of miR-150 promotes NSCLC cell proliferation and migration. However, the potential mechanisms of miR-150-5p regulation of CRC progression remain unclear.

In this study, we found that miR-150-5p was obviously downregulated in CRC tissues and negatively correlated with TNM stage, pN stage and overall survival (OS). Furthermore, we identified vascular epithelial growth factor A (VEGFA), a member of the VEGF family, as a direct target gene of miR-150-5p. We also provided evidence that miR-150-5p inhibited tumor growth, metastasis and angiogenesis in CRC. Lastly, we verified that miR-150-5p suppressed CRC initiation and progression via VEGFA/VEGFR2/Akt/mTOR signaling.

## RESULTS

### miR-150-5p is downregulated in CRC tissues and cell lines

**Figure 1:**
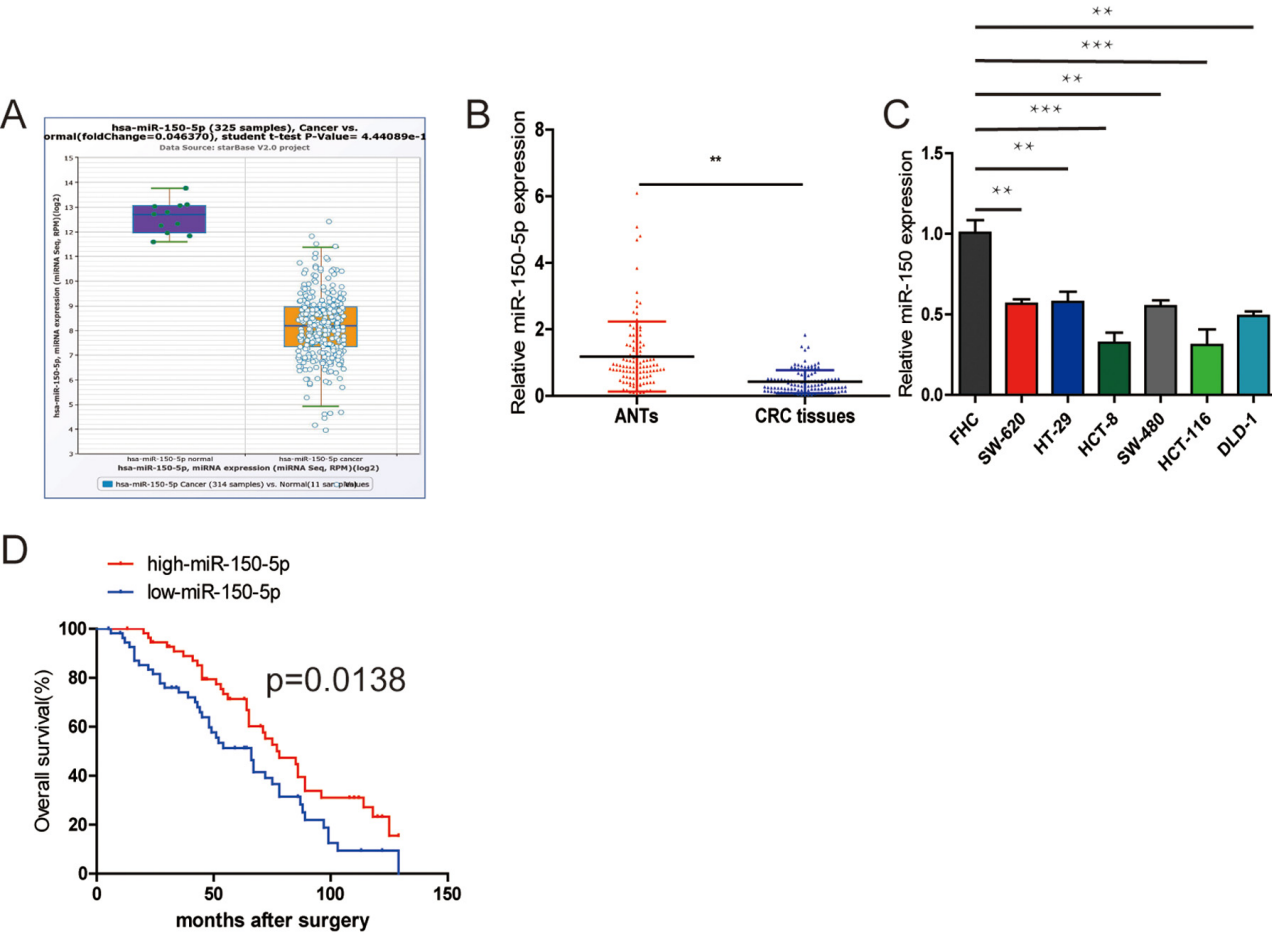
**Downregulation of miR-150-5p in CRC and its association with poor OS.** (**A**) miR-150-5p was found to be downregulated compared with normal tissues in the Starbase database. (**B**) Quantitative RT-PCR was performed to detect the relative expression of miR-150-5p in CRC tissues compared with ANTs. (**C**) The relative expression of miR-150-5p was assessed in different CRC cell lines and FHC using qRT-PCR. (**D**) Kaplan-Meier survival analysis was employed to detect the correlation between the levels of miR-150-5p expression and overall survival of CRC patients. Data were shown as mean±SD. **p<0.01.

First, to determine the clinical significance of miR-150-5p in patients with CRC, we used the StarBase database (http://starbase.sysu.edu.cn/) to reveal that miR-150-5p expression was downregulated in CRC tissues compared with normal tissues (Fig. 1A) and the database is accompanied with gene expression data of 32 types of cancers which are derived from 10,882 RNA-seq and 10,546 miRNA-seq data. Then, we measured the miR-150-5p expression in 112 pairs of CRC tissues using qRT-PCR. The results showed that miR-150-5p was significantly downregulated in CRC tissues compared with that in matched adjacent normal tissues (ANTs) (Fig. 1B). In addition, the miR-150-5p expression was markedly downregulated in all CRC cell lines in comparison with colonic epithelial cell (FHC) (Fig. 1C). Next, we examined the potential clinical significance of miR-150-5p in CRC. We used the median miR-150-5p expression as a cut-off value. As shown in Table 1, low miR-150-5p expression was markedly associated with TNM stage (*p*=0.013) and pM stage (*p*=0.031).

**Table 1:** Correlation between miR-150-5p expression and different clinical characteristics.

Characteristics	n=112	miR-150-5p expression		*P* value
		Low(%)(n= 56)	High(%)(n=56)	
Gender				0.110
Male	74	33	41	
Female	38	23	15	
Age(years)				0.106
<60	36	14	22	
≥60	76	42	34	
Tumor location				0.570
colon	59	31	28	
rectal	53	25	28	
TNM stage				0.013
I-II	65	26	39	
III-IV	47	30	17	
Differentiation				0.114
Low	27	10	17	
Moderate	70	37	33	
High	15	9	6	
PN stage				0.819
N0	66	33	33	
N1	31	14	17	
N2	15	9	6	
pM stage				0.031
M0	96	44	52	
M1	16	12	4	
Tumor size				0.701
<5cm	66	34	32	
≥5cm	46	22	24	
CEA				0.115
<10ng/ml	72	32	40	
≥10ng/ml	40	24	16	
CA199				0.088
<10U/ml	51	21	30	
≥10U/ml	61	35	26	

The Kaplan-Meier survival analysis indicated that CRC patients with low miR-150-5p had an obviously shorter overall survival (OS) (Fig. 1C). Furthermore, the results of univariate and multivariate analyses of potential prognostic factors for overall survival are shown in Table 2. The univariate analysis indicated that TNM stage (*p*=0.001), pN stage (*p*=0.006), pM stage (*p*=0.002) and miR-150-5p (*p*=0.004) were predictors of OS. The multivariate analysis also showed that higher TNM stage (*p*=0.006), pN stage (*p*=0.017) and pM stage (*p*=0.008), as well as lower miR-150-5p (*p*=0.011), were significantly correlated with poor OS (Table 2). Based on these results, we suggested that miR-150-5p might play a key role in CRC progression.

**Table 2:** Results of the univariate and multivariate analyses of prognostic factors for overall survival in patients with CRC.

Characteristics	Multivariate analysis for OS		Univariate analysis for OS		
	HR(95% CI)	*P*	HR(95% CI)	*P*	
Gender(male/female)	—	—	1.158(0.739-1.812)		0.522
Age(<60/≥60)	—	—	0.928(0.618-1.551)		0.928
Tumor location(colon/rectal)	—	—	1.160(0.754-1.785)		0.500
TNM stage(I-II/III-IV)	1.833(1.092-3.019)	0.006	2.073(1.339-3.209)		0.001
Differentiation(low/moderate/high)	—	—	1.322(0.935-1.871)		0.114
PT stage(T1-T2/T3-T4)	—	—	0.674(0.357-1.271)		0.223
PN stage(N0/N1/N2)	1.654(1.104-2.746)	0.017	1.814(1.182-2.785)		0.006
PM stage(M0/M1)	2.745(1.432-5.713)	0.008	2.702(1.451-5.031)		0.002
Tumor size(<5cm/≥5cm)	—	—	0.654(0.420-1.018)		0.060
CEA(<10ng/ml/≥10ng/ml)	—	—	0.775(0.479-1.192)		0.228
CA199(<10U/ml/≥10U/ml)	—	—	0.751(0.488-1.156)		0.193
miR-150-5p(low/high)	1.431(1.091-2.615)	0.011	1.923(1.213-2.918)		0.004

Abbreviations: CI, confidence interval; HR, hazard ratio.

### miR-150-5p inhibits cell proliferation, migration, invasion and angiogenesis in vitro

**Figure 2:**
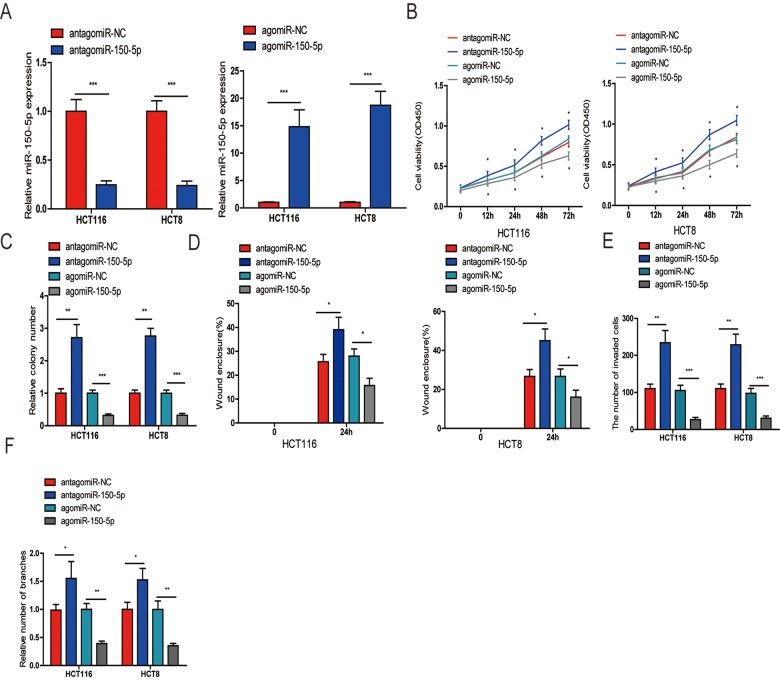
**miR-150-5p suppressed CRC cell proliferation, migration, invasion and HUVEC tube formation in vitro.** (**A**) qRT-PCR was used to detect the expression of miR-150-5p in CRC cells transfected with antagomiR-150-5p or agomiR-150-5p and their negative control (antagomiR-NC, agomiR-NC). (**B**, **C**) CCK-8 (**B**) and colony formation (**C**) were performed to evaluate the proliferation abilities of CRC cells transfected with antagomiR-150-5p, agomiR-150-5p or negative control (antagomiR-NC, agomiR-NC). (**D**, **E**) Wound-healing (**D**) and transwell (**E**) assays were employed to detect the ability of migration and invasion of antagomiR-150-5p or agomiR-150-5p-transfected CRC cells and their negative control. (**F**) HUVECs were cultured in TCM derived from CRC cells transfected with antagomiR-150-5p or agomiR-150-5p and their negative control in 24-well Matrigel-coated plates. Each bar represents the mean ± SD of three independent experiments. *p<0.05, **p<0.01, ***p<0.001.

To determine the biological function of miR-150-5p in CRC, we selected HCT116 and HCT8 cells for transfection of agomiR-150-5p and antagomiR-150-5p or their corresponding negative controls (agomiR-NC and antagomiR-NC). The efficiency of transfection was confirmed by qRT-PCR (Fig. 2A). First, a CCK-8 assay performed to assess CRC cell growth showed that upregulation of miR-150-5p resulted in decreased cell proliferation, while the inhibition of miR-150-5p expression obviously increased cell proliferation in HCT116 and HCT8 cells (Fig. 2B). A colony formation assay further substantiated the role of miR-150-5p in inhibiting the proliferation of CRC cells (Figs. 2C and Fig. S1A). Next, wound-healing and transwell invasion assays were performed in HCT116 and HCT8 cells. As shown in Figs. 2D and Fig. S1B, Figs 2E and Fig. S1C, the migratory and invasive abilities were significantly reduced when miR-150-5p expression was increased, while miR-150-5p downregulation significantly increased cell migration and invasion in HCT116 and HCT8 cells. Additionally, to explore whether miR-150-5p was a key angiogenesis suppressor in CRC, we investigated the influence of miR-150-5p on HUVEC tube formation and showed that miR-150-5p upregulation obviously reduced the tube-formation capacity, while silencing miR-150-5p increased the tube-formation ability of HUVECs (Fig. 2F and Fig. S1D).

### miR-150-5p ameliorates CRC growth, metastasis and angiogenesis in vivo

**Figure 3:**
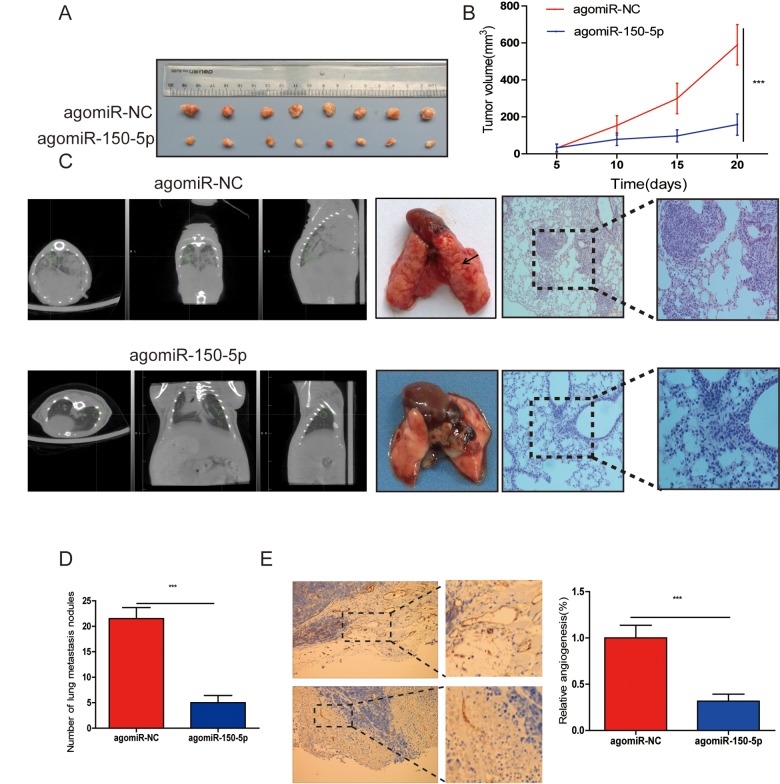
**miR-150-5p inhibited tumor growth, metastasis and angiogenesis in vivo.** (**A**) Photographs of nude mice and xenograft tumors of CRC cells with miR-150-5p overexpression or not after 20 days of implantation. (**B**) Growth curves of xenografts of CRC cells with or without miR-150-5p overexpression. (**C**) The representative images of lungs from nude mice (left), representative CT scans of lung metastatic foci of the nude mice (middle) and the microscopic images of lung tissue sections stained by hematoxylin and eosin (right). (**D**) A statistical plot of the average number of lung metastases in the tail vein injection. (**E**) These tumor tissues were stained using anti-CD31 antibody, data are shown as the mean ±SD. n=8. ***p<0.001.

To evaluate the biological functions of miR-150-5p in vivo, CRC cells were subcutaneously injected into nude mice. First, we investigated the effects of miR-150-5p on the xenograft growth in vivo and found that the volume of tumors injected with agomiR-150-5p was significantly smaller than that with agomiR-NC (Fig. 3A, 3B). Furthermore, to examine the influence that miR-150-5p had on metastasis in vivo, CRC cells were injected into nude mice and agomiR-150-5p or agomiR-NC via the tail vein. After 8 weeks, computed tomography (CT) scanning was used on each mouse, and results revealed that the metastatic lesions in the agomiR-150-5p group were less abundant than that in the agomiR-NC group (Fig. 3C). Following CT scanning, these mice were sacrificed, and hematoxylin-eosin (HE) was employed to stain mouse lungs. Compared with low miR-150-5p-expressing CRC cells, upregulation of miR-150-5p significantly decreased the number of metastatic nodules (Fig. 3C, 3D). We then detected CD31 (a marker for microvessels denoting enhanced angiogenesis) expression using immunohistochemistry (IHC) in xenografts tissues and showed that CD31 expression in the agomiR-150-5p group was significantly lower than those in agomiR-NC group (Fig. 3E).

### VEGFA is a direct target gene of miR-150-5p

**Figure 4:**
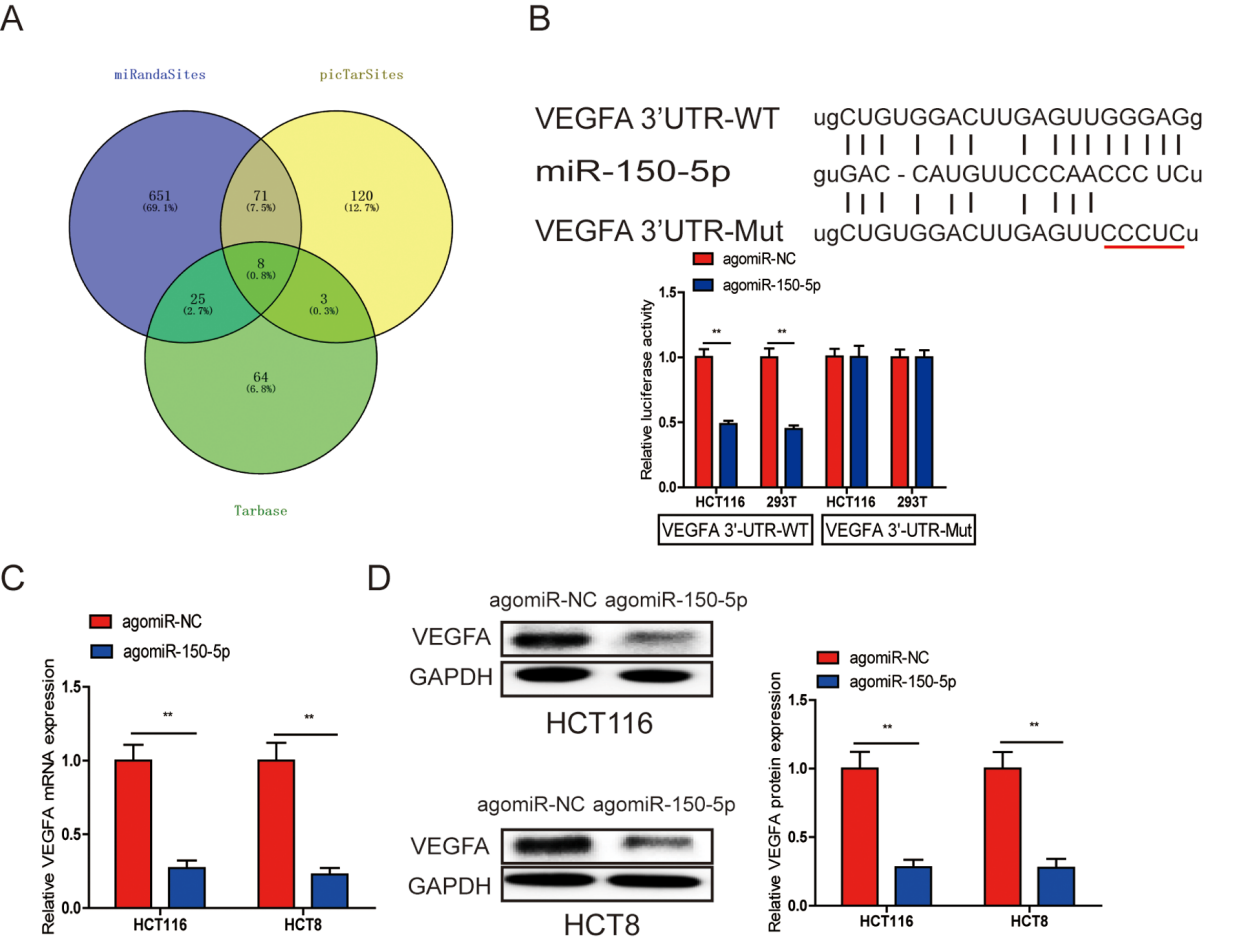
**A. VEGFA was a direct target of miR-150-5p in CRC.** (**A**) The direct target genes of miR-150-5p were predicted using the PicTarSites, miRandaSites and Tarbase databases. (**B**) Wild-type and mutant VEGFA-3’UTR sequences were cloned into luciferase reporter. Luciferase activity was determined in HCT116 and 293T cells cotransfected with agomiR-150-5p or agomiR-NC and pmirGLO-VEGFA-3’UTR-WT or pmirGLO-VEGFA-3’UTR-Mut. Luciferase activities were normalized to that of renilla luciferase. C, D. qRT-PCR (**C**) and western blot (**D**) analyses showed that both VEGFA mRNA and protein expression levels were dramatically suppressed by agomiR-150-5p in HCT116 and HCT8 cells, GAPDH was used as the internal control. **p<0.01.

Many miRNAs have been reported to be dysregulated and exert their functions mainly through their target genes [13,14]. The PicTarSites, miRandaSites and Tarbase databases were used to search for potential target genes of miR-150-5p. As shown in Fig. 4A, eight potential genes were predicted in all four databases. VEGFA, which was selected as a target gene of miR-150-5p, is closely associated with metastasis and angiogenesis of human cancers. Next, dual-luciferase reporter assays were used to determine whether VEGFA was a direct target gene in CRC cells. The 3’-UTR of VEGFA wild-type (WT-VEGFA-3’UTR), as well as VEGFA mutant type (Mut-VEGFA-3’UTR) were constructed and cloned into pmirGLO luciferase reporter, then cotransfected with agomiR-150-5p or agomiR-NC into HCT116 and 293T cells. Luciferase reporter assay confirmed that miR-150-5p overexpression obviously reduced the luciferase activity of pmirGLO-VEGFA-3’UTR-WT but showed no significant effect on the luciferase activity of pmirGLO-VEGFA-3’UTR-Mut (Fig. 4B). Additionally, qRT-PCR and Western blot analyses showed that both VEGFA mRNA and protein levels are dramatically decreased after miR-150-5p upregulation in HCT116 and HCT8 cells (Fig. 4C, 4D).

### VEGFA upregulation could reverse the inhibitory effect caused by miR-150-5p

**Figure 5:**
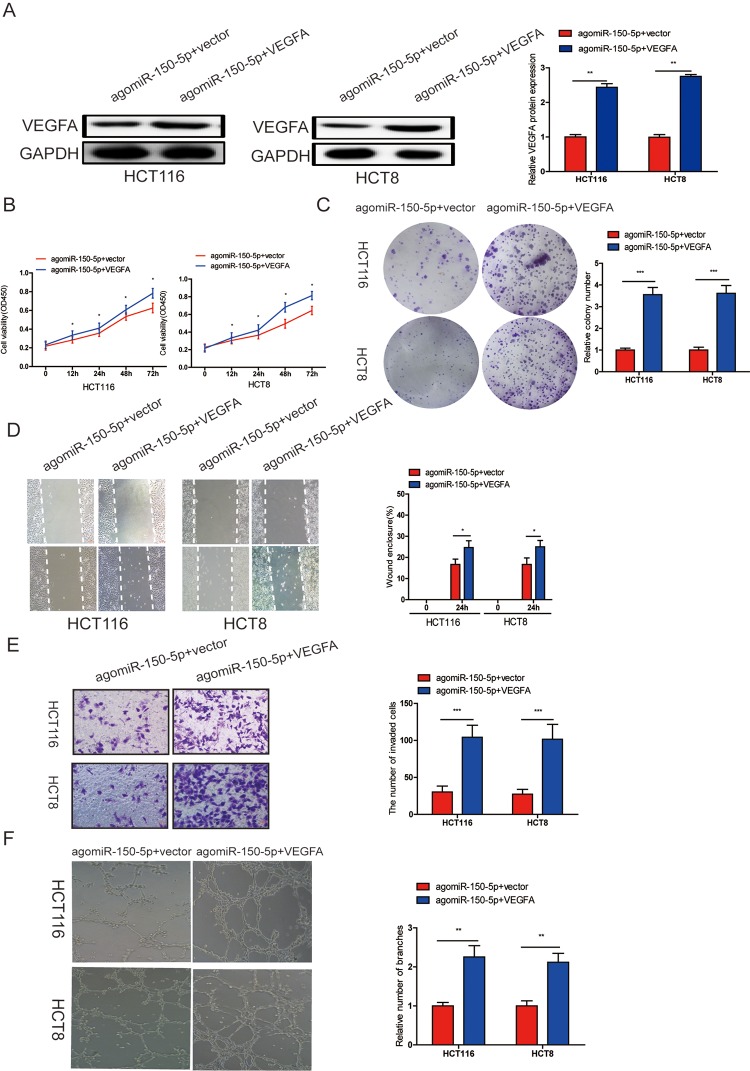
**miR-150-5p inhibited CRC progression by targeting VEGFA.** (**A**) VEGFA protein expression was determined in HCT116 and HCT8 cells transfected with agomiR-150-5p with VEGFA expression plasmid or empty vector using western blot; GAPDH was used as the internal control. (**B**-**D**) Cell proliferation (**B**, **C**), migration (**D**)and invasion (**E**) were evaluated in HCT116 and HCT8 cells transfected with agomiR-150-5p with VEGFA expression plasmid or empty vector. (**F**) HUVECs were cultured in TCM derived from HCT116 and HCT8 cells transfected with agomiR-150-5p plus VEGFA expression plasmid or empty vector. Data are shown as the mean±SD. *p<0.05, **p<0.01, ***p<0.001.

To investigate whether miR-150-5p suppressed proliferation, migration, invasion, angiogenesis and EMT process through VEGFA, we cotransfected agomiR-150-5p and pcDNA 3.1-VEGFA or empty vector, and VEGFA upregulation was verified by western blotting (Fig. 5A). Through CCK-8, colony formation, wound-healing, transwell invasion and tube formation assays, we found that ectopic VEGFA expression could partially reverse the suppression of proliferation (Fig. 5B, 5C), migration (Fig. 5D), invasion (Fig. 5E) and angiogenesis (Fig. 5F) caused by miR-150-5p overexpression. These data indicated that VEGFA was not only a direct target gene but also a functional mediator of miR-150-5p in CRC.

### The expression of VEGFA is negatively correlated with miR-150-5p

**Figure 6:**
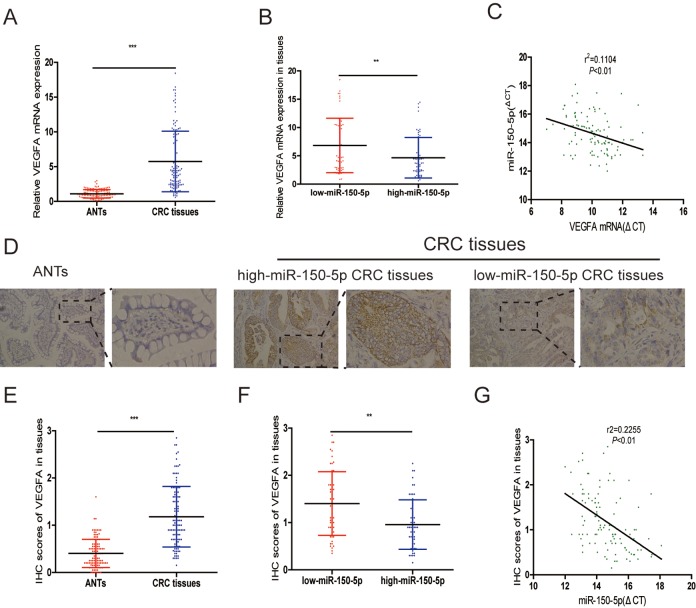
**VEGFA was reversely correlated with miR-150-5p in CRC tissues.** (**A**) qRT-PCR analysis of VEGFA mRNA expression in CRC tissues and matched adjacent normal tissues. (**B**) Scatterplots of the average VEGFA mRNA expression in patients with low or high expression of miR-150-5p in CRC tissues. (**C**) The correlation between miR-150-5p levels and VEGFA mRNA levels. (**D**) Representative images of IHC in adjacent normal tissues, low or high miR-150-5p CRC tissues. (**E**) The statistical graph showed that the IHC score of VEGFA in CRC tissues was significantly higher than in matched adjacent normal tissues. (**F**) Scatterplots of the average staining scores for VEGFA expression in patients with low or high expression of miR-150-5p. G. The correlation between miR-150-5p levels and VEGFA protein levels. Data were shown as the mean±SD. **p <0.01, ***p<0.001.

qRT-PCR performed to detect VEGFA mRNA in CRC tissues clearly showed that VEGFA mRNA was overexpressed in CRC tissues compared with matched adjacent normal tissues (ANTs) (Fig. 6A), moreover, we analyzed VEGFA mRNA in CRC tissues at different miR-150-5p levels. The low versus high miR-150-5p levels were defined at the median of miR-150-5p level. The levels of VEGFA mRNA expression in high-miR-150-5p CRC tissues were markedly lower than that in low-miR-150-5p CRC tissues (Fig. 6B). Meanwhile, an obvious negative correlation was found between miR-150-5p and VEGFA mRNA (Fig. 6C).

In addition, IHC was performed to assess the protein expression of VEGFA in CRC tissues, and as shown in Fig. 6D and 6E, the IHC staining intensity indicated that the VEGFA protein expression levels in CRC tissues were obviously higher than in adjacent normal tissues. The VEGFA was relatively upregulated in the low-miR-150-5p expression CRC tissues compared with in high-miR-150-5p CRC tissues (Fig. 6D and 6F); a negative correlation between VEGFA and miR-150-5p was also noted in CRC tissues (Fig. 6G).

### VEGFA knockdown significantly inhibits CRC progression

**Figure 7:**
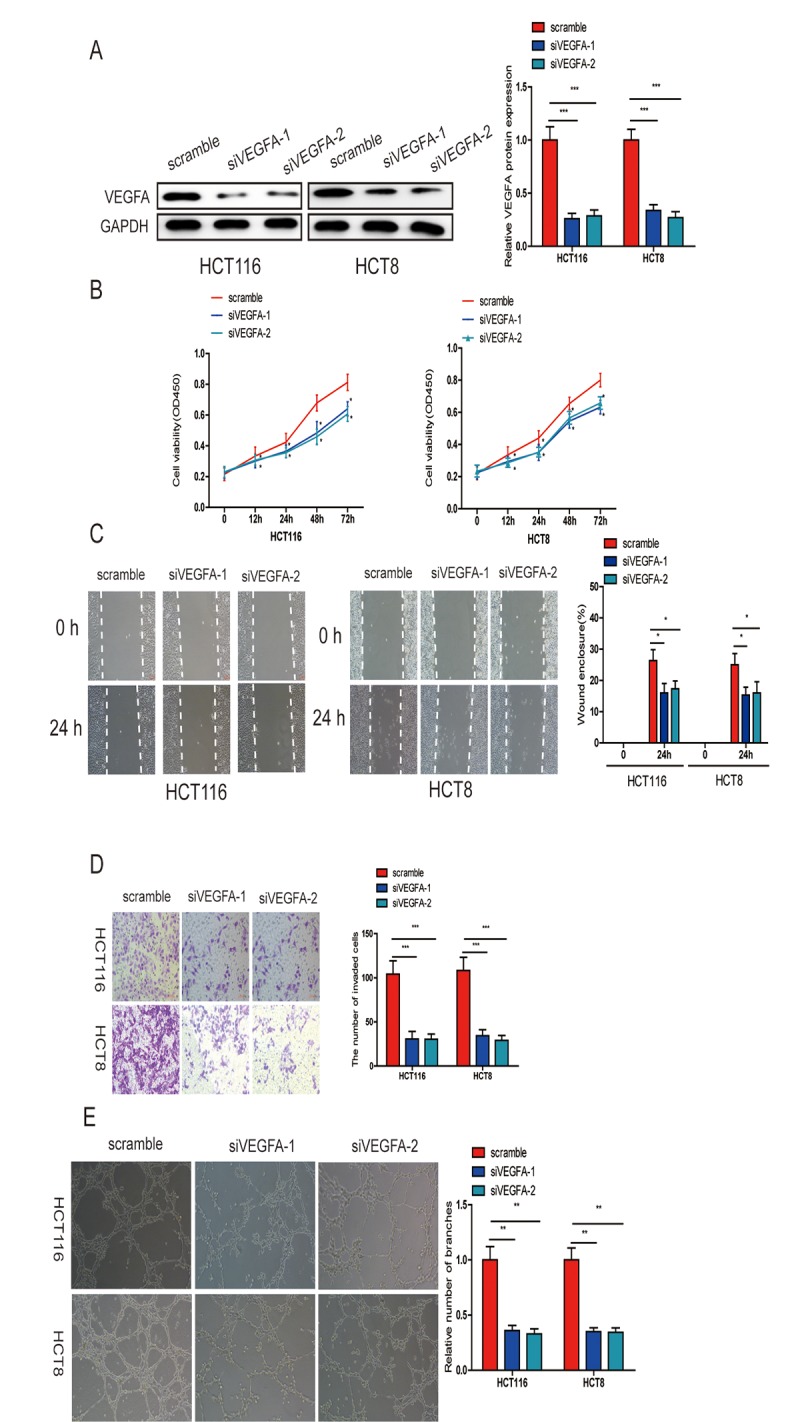
**VEGFA knockdown significantly inhibited CRC progression.** (**A**) VEGFA expression was downregulated in HCT116 and HCT8 cells transfected with siVEGFA-1 or siVEGFA-2. (**B**) VEGFA knockdown inhibited CRC cell proliferation (**B**), migration (**C**), invasion (**D**) and HUVECs tube formation (**E**). Data are shown as the mean±SD of three independent experiments. *p<0.05, **p<0.01, ***p<0.001.

To further verify that miR-150-5p exerts its inhibitory effect via VEGFA, we knocked down VEGFA in HCT116 and HCT8 cells (Fig. 7A) and found that this markedly suppressed CRC cell growth, migration, invasion and HUVEC tube formation (Fig. 7B-7E). These data further proved that miR-150-5p exerted its biological function by inhibiting VEGFA expression.

### miR-150-5p inactivates the Akt/mTOR signaling pathway by directly inhibiting VEGFA

**Figure 8:**
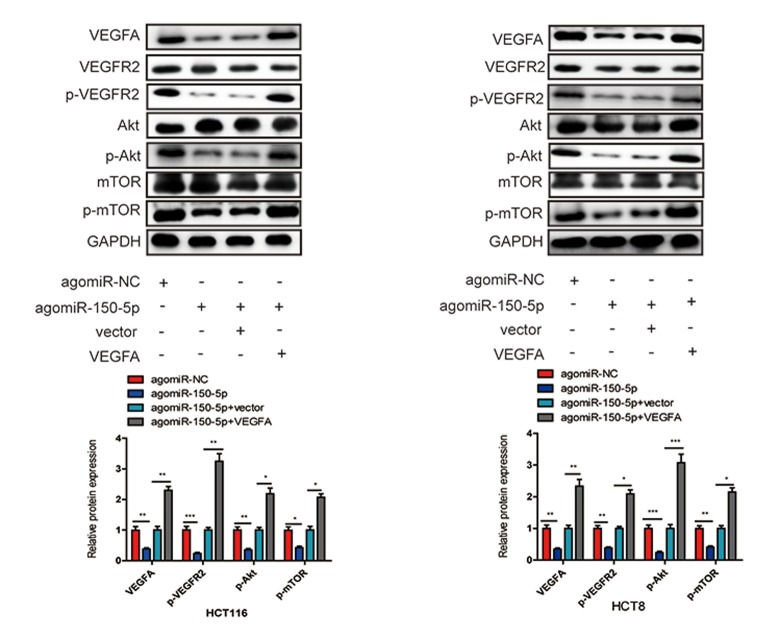
**miR-150-5p inhibited VEGFA/VEGFR2/Akt/mTOR signaling pathway in CRC.** Western blot was used to measure the expression of VEGFA, VEGFR2, p-VEGFR2, Akt, p-Akt, mTOR, p-mTOR in transfected HCT116 and HCT8 cells. GAPDH was used as a loading control. Data are shown as the mean±SD of three independent experiments. *p<0.05, **p<0.01, ***p<0.001.

Accumulating evidence has shown that VEGFA could bind to its receptor VEGFR2, then activate PI3K and its downstream targets Akt and mTOR, which play crucial roles in cancer progression and survival [15–17]. To further explore whether miR-150-5p inhibited CRC development through VEGFA-mediated Akt/mTOR signaling pathway, we found that miR-150-5p overexpression significantly decreased the level of VEGFA, and then significantly suppressed the phosphorylation of VEGFR2 (p-VEGFR2), Akt (p-Akt) and mTOR (p-mTOR) in HCT116 and HCT8 cells. Next, we treated miR-150-5p in HCT116 and HCT8 cells with VEGFA expression plasmid and found that VEGFA treatment could at least partially increase the expression of p-VEGFR2, p-Akt and p-mTOR (Fig. 8). These results revealed that miR-150-5p exerted its inhibitory function via the VEGFA/VEGFR2/Akt/mTOR signaling pathway.

## DISCUSSION

Many studies have demonstrated that dysregulated miRNAs are involved in tumor initiation and progression, including CRC [18]. In our study, we sought to detect an association between miR-150-5p and a clinical outcome in CRC and showed that miR-150-5p was closely associated with TNM stage, distant metastasis and overall survival.

Yushuai Mi, et al. [19] have reported that miR-181a-5p was dysregulated in gastric cancer tissues and cell lines, and high miR-181a-5p expression predicted poor survival in patients with gastric cancer. In our study, we found that miR-150-5p was downregulated in CRC tissues and cell lines. These data indicate that miR-150-5p may play a tumor suppressive role in CRC.

Recently, Mohamed Salem, et al. [20] verified that miR-590-3p promoted ovarian cancer (EOC) growth and metastasis in vitro and vivo. Yuan Gu, et al. [21] reported that miR-191, which has been extensively studied in various types of cancers, suppressed angiogenesis by activating NF-ĸB signaling. Here, we validated that miR-150-5p overexpression inhibited CRC cell proliferation, migration, invasion and angiogenesis in vitro and in vivo, while decreased miR-150-5p promoted CRC progression in vitro.

It is well-accepted that miRNAs exert their function by modulating their target genes by elevating translational repression or mRNA degradation [22,23]. Wei Kang, et al. [24] have reported that miR-375 inhibited tumor growth in vitro and in vivo, and its targets, YAP1, TEAD4 and CTGF, could partially abolish the tumor-suppressive effect in gastric cancer. Yue Yu, et al*.* [25] have reported that miR-190 overexpression suppressed EMT and breast cancer progression, and SMAD2, a target of miR-190, could abolish the effects of miR-190 on breast cancer cell EMT and invasion. Herein, using a luciferase reporter assay, we demonstrated that VEGFA was a direct target of miR-150-5p. Moreover, we found that VEGFA upregulation could reverse the inhibitory effect caused by miR-150-5p overexpression, whereas VEGFA knockdown could also inhibit CRC cell growth, migration, invasion and HUVEC tube formation. Additionally, we also showed that the expression of miR-150-5p was reversely correlated with VEGFA expression in CRC tissues. Based on these findings, we could conclude that miR-150-5p exert its biological function by targeting VEGFA in CRC.

Akt and mTOR are both downstream targets of VEGFA. Previous studies have reported that the Akt/mTOR signaling pathway played a critical role in various biological processes of cancers, such as proliferation, metastasis, survival and angiogenesis [26–28].We further examined whether miR-150-5p inhibited tumor initiation and progression by inactivating the VEGFA/VEGFR2/Akt/mTOR signaling pathway, and our data showed that miR-150-5p overexpression could significantly reduce the expression of VEGFA and reduce the activity of VEGFR2 and the downstream Akt/mTOR signaling pathway.

In summary, our study demonstrated the pivotal roles of the miR-150-5p-mediated

VEGFA/VEGFR2/Akt/mTOR signaling pathway on the tumorigenesis and progression of CRC. This research not only provides a new insight into the mechanism of CRC progression but also highlights miR-150-5p as a potential diagnostic biomarker and therapeutic target of CRC.

## MATERIALS AND METHODS

### Patient samples

One hundred-twelve fresh CRC tissues and matched adjacent normal tumor tissues were obtained from CRC patients who received radical surgery at Nanjing First Hospital, Nanjing Medical University, between January 2001 and December 2007. The patients who received systemic treatment were excluded. All sample tissues were stored at -80°C until RNA extraction and embedding in paraffin. Informed consents were obtained from all CRC patients, and this study was approved by the Institutional Review Board of Nanjing First Hospital, Nanjing Medical University. Clinicopathologic characteristics of these patients are listed in Table 1.

### Cell culture

The human CRC cell lines including HCT116, HCT8, HT29, SW620, SW480 and DLD-1 and normal colonic epithelial cells (FHC) were obtained from ATCC. These cells were cultured in Dulbecco’s Modified Eagle Medium (DMEM, Gibco, USA) with 100 U/ml penicillin, 0.1 mg/ml streptomycin and 10% fetal bovine serum (FBS, Gibco, USA) at 37°C in 5% CO_2_ atmosphere.

### Cell transfection

AgomiR-150-5p, antagomiR-150-5p, small interference RNA targeting VEGFA (siVEGFA-1, siVEGFA-2) and their corresponding negative controls (agomiR-NC, antagomiR-NC and scrambled sequence) were obtained from GenePharma (Shanghai, China), and their sequences are listed in Supplementary Materials and Methods. pcDNA3.1-VEGFA and empty vector were obtained from GeneCreate (Wuhan, China). Cell transfections were performed using Lipofectamine^TM^ 2000 (Invitrogen, USA) following the manufacturer’s protocol.

### Cell proliferation assays

Cell proliferation assays were performed using Cell Counting Kit (CCK-8, KeyGEN BioTECH, China) in accordance with the manufacturer’s instructions. Approximately 1×10^4^ transfected cells were seeded into a 96-well plate. After 12, 24, 48 and 72 h, 10 µl CCK-8 test solution was added into a 96-well plate and incubated for 3 h. The absorbance at 450 nm was measured in a microplate reader (Infinite M200 Pro, Tecan).

### Wound-healing assays

HCT116 and HCT8 cells were seeded into 6-well plates and grew to reach 90% confluency. Then, cells were scraped using 200-µl pipette tips, and the wound closure was detected using an inverted microscope (Nikon, Japan) at 100× magnification.

### Transwell invasion assays

The cell invasion was evaluated by transwell assay. For this, 3×10^5^ cells were cultured in 200 µl of serum-free media in the upper chamber precoated with 100 µl 2% Matrigel (BD Biosciences, USA). The lower chamber was filled with 500 µl of medium containing 10% FBS. After incubation for 36 h, the noninvaded cells were wiped off in the upper chamber with cotton swabs, and the cells in the lower surface were fixed by methanol, then stained with 0.1% crystal violet (Beyotime, China). The invaded cells were counted under inverted microscope (Nikon, Japan) at 200× magnification.

### Tube formation assays

HUVECs cells (7×10^5^) were suspended in the mixture of tumor-conditioned medium (TCM, 300 µl) and DMEM containing 10% FBS (300 µl), and then plated into a 24-well plate precoated with Matrigel (200 µl per well, BD Biosciences, USA). Tube formation was observed after 6 h incubation at 37°C and was imaged with a computer-assisted inverted microscope (Nikon, Japan), the number of tube branches was counted using Image J software.

### Dual-luciferase reporter assays

The 293T and HCT116 cells were cotransfected with pmirGLO-VEGFA-3’UTR-WT and pmirGLO-VEGFA-3’UTR-Mut reporter plasmids and agomiR-NC and agomiR-150-5p. Twenty-four hours after transfection, dual-luciferase reporter assay (Promega, Madison, WI, USA) was performed to measure the relative luciferase activity normalized to renilla luciferase activity.

### Western blot

The total proteins were extracted from cells with RIPA lysis buffer mixed with phenylmethyl sulfonylfluoride (PMSF), protein inhibitors and phosphatase inhibitors (KeyGEN BioTECH, China). Equal amounts of proteins were separated with 10% SDS-PAGE gel and transferred to polyvinylidene difluoride (PVDF) membranes (Millipore, USA). The membranes were blocked with 5% bovine serum albumin (BSA) for 1.5 h and then incubated with primary antibodies: rabbit polyclonal anti-VEGFA (1:1000, ab46154, abcam, UK), anti-VEGFR2 (1:1000, ab39256, abcam, UK), anti-phospho(Y1175)-VEGFR2 (1:1000, ab194806, abcam, UK), anti-Akt (#9272, Cell Signaling Technology, USA), anti-phospho (Ser473)-Akt (1:2000, #4060, Cell Signaling Technology, USA), anti-mTOR (1:1000, ab2732, abcam, UK), anti-phospho(S2448)-mTOR (1:1000, ab84400 abcam, UK) and rabbit anti-GAPDH (1:10000, ab9485, abcam, UK). Proteins were then detected by enhanced chemiluminescence system (ECL) reagent (KeyGEN BioTECH, China) after incubation with secondary antibodies for 1 h at room temperature.

### RNA isolation and quantitative RT-PCR

TRIzol reagent (Invitrogen, USA) was used to extract total RNA in accordance with manufacturer’s instructions. MiR-150-5p expression was detected by a Hairpin-it^TM^ microRNA and U6 snRNA normalization RT-PCR quantitation kit (Genepharma, China). For VEGFA mRNA, complementary DNA (cDNA) was synthesized using PrimeScript^TM^ reagent kit with gDNA Eraser (Takara, Dalian, China) and qRT-PCR was analyzed using SYBR Premix Ex Taq kit (Takara, Dalian, China). The relative expression of miRNA or mRNA was analyzed using the 2^-ΔΔCT^ method. All results were normalized to GAPDH or U6 gene. The primers for VEGFA and GAPDH mRNA are listed below (Table 3):

**Table 3:** The primers for VEGFA and GAPDH mRNA.

VEGFA	Forward: 5’-TGGCTCACTGGCTTGCTCTA-3’
	Reverse: 5’-ATCCAACTGCACCGTCACAG-3’
GAPDH	Forward: 5’-GGTGGTCTCCTCTGACTTCAA-3’
	Reverse: 5’-GTTGCTGTAGCCAAATTCGTTGT-3’

### Immunohistochemistry

The CRC tissues, adjacent normal tissues and xenograft tissues were collected, paraffin-embedded and cut into 4-µm-thick sections. Sections were incubated with rabbit anti-VEGFA and anti- CD31 antibodies at 4°C overnight, followed by incubation with secondary antibodies. The sections were visualized under an inverted microscope (Nikon, Japan) at 200× or 400× magnification. The staining intensity was scored by two independent pathologists into the following four categories: no staining=0, weak staining=1, moderate staining=2 and strong staining=3. The stain-positive tissues were scored into four grades: 0 (0%), 1 (1-33%), 2 (34%-66%), and 3 (67%-100%). The final ICH score was calculated by multiplying the percentage of positive cells by the intensity score.

### In vivo experiments

All animal experiments were approved by the Animal Care Committee of Nanjing First Hospital, Nanjing Medial University (acceptance No. SYXK 20160006). For xenografted tumor model, 1×10^7^ CRC cells in 0.2 ml PBS were subcutaneously injected into BABL/c nude mice, which were randomly divided into two group (n=8 per group). After tumor formation (day 6), 2 nmol agomiR-150-5p or agomiR-NC were injected into tumors, and the injections were performed 7 times at an interval of 2 days between each injection (day 6, 8, 10, 12, 14, 16, 18). The mice were sacrificed 2 days after their last injection, and the tumor volumes were calculated with the following equation:

V=0.5 × (length × width^2^)

For metastasis experiments, 2×10^6^ cells in 0.2 ml PBS were injected into the tail vein of nude mice which were randomly divided into two groups (n=8 per group). After 8 weeks of injection, computed tomography (CT) scans were performed to detect the lung metastasis in mice. After CT scans, mice were sacrificed, and their lungs were removed and stained by hematoxylin and eosin (HE) Staining.

### Statistical analysis

All experiments were performed more than three times. The data are expressed as the mean ± SD and analyzed by SPSS 22.0 software. The Student’s t-test and one-way analysis of variance (ANOVA) were performed to detect the differences between groups. Pearson’s, Mann-Whitney U-test or χ2-test was used to analyze the relationship between miR-150-5p expression and clinicopathological features. The Kaplan-Meier method was applied to assess overall survival (OS). The survival curves were compared with log-rank test. The follow-up time was censored if the patient was lost` during follow-up. Cox proportional hazards model was used to perform multivariate analysis and calculate the 95% confidence interval (95% CI). A statistically significant difference was considered at *P* < 0.05.

## Supplementary Material

Supplementary Figure

Supplementary Materials and Methods
