# Correction for: miR-150-5p suppresses tumor progression by targeting VEGFA in colorectal cancer

**DOI:** 10.18632/aging.203069

**Published:** 2021-05-14

**Authors:** Xiaoxiang Chen, Xueni Xu, Bei Pan, Kaixuan Zeng, Mu Xu, Xiangxiang Liu, Bangshun He, Yuqin Pan, Huiling Sun, Shukui Wang

**Affiliations:** 1 General Clinical Research Center, Nanjing First Hospital, Nanjing Medical University, Nanjing 210006, Jiangsu, China; 2 Medical College, Southeast University, Nanjing 210009, Jiangsu, China

**Keywords:** correction

Original article: Aging. 2018; 10:3421–3437. . https://doi.org/10.18632/aging.101656

**This article has been corrected:** The authors replaced panel D of Figure 7, where the images of HCT116/siVEGFA-1 and HCT116/siVEGFA-2 were identical, because the image HCT116/siVEGFA-1 was accidentally used twice. The correct image of HCT116/siVEGFA-2 from the original set of experiments was used for new panel 7D, which is provided below.

The authors replaced panel B of Supplementary Figure 1, where the images of HCT8/agomiR-NC (0 h) group and agomiR-150-5p+vector (24h) group were used instead of the images of HCT8/agomiR-150-5p group (0, 24h). The correct images of HCT8/agomiR-150-5p group (0, 24h) from the original set of experiments were used for new panel S1B. Additionally, authors added 0 and 24h marks to the panel S1B, which is provided below.

Authors deeply apologize for these errors and testify that these alterations do not affect the results or conclusions of this work.

**Figure 7:**
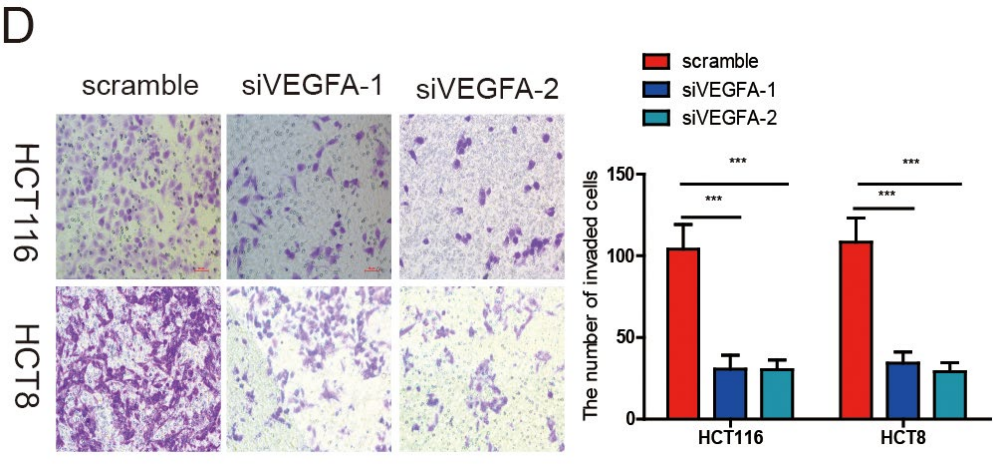
**VEGFA knockdown significantly inhibited CRC progression.** (**A**) VEGFA expression was downregulated in HCT116 and HCT8 cells transfected with siVEGFA-1 or siVEGFA-2. (**B-E**) VEGFA knockdown inhibited CRC cell proliferation (**B**), migration (**C**), invasion (**D**) and HUVECs tube formation (**E**). Data are shown as the meanv±SD of three independent experiments. *p<0.05, **p<0.01, ***p<0.001.

**Supplementary Figure S1:**
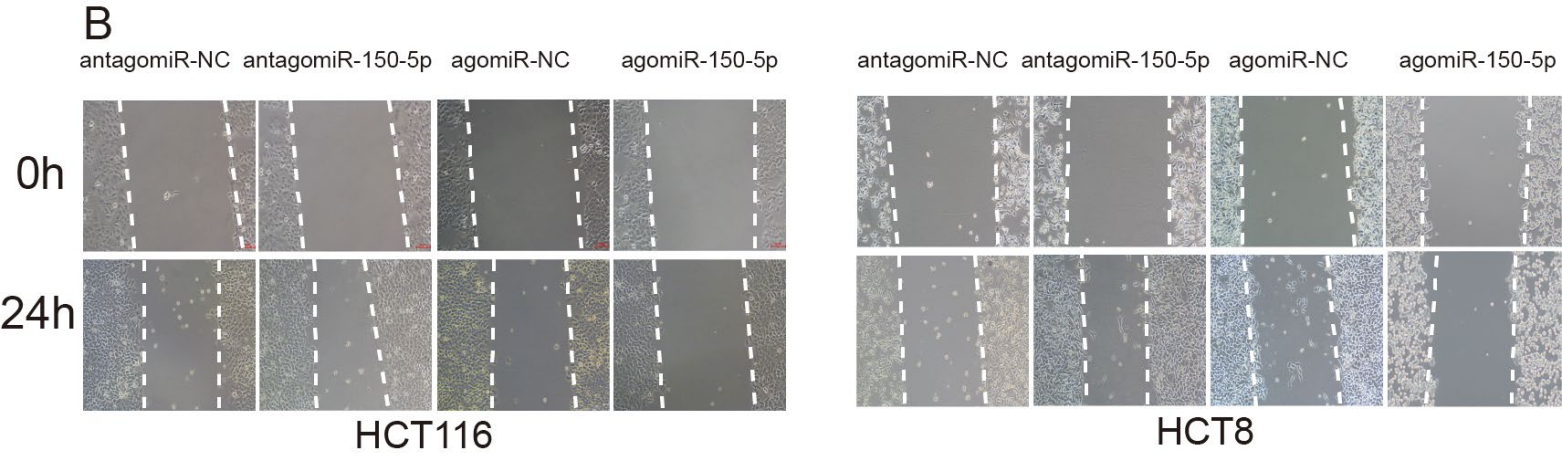
(**A-D**) Representative images of colony formation (**A**), wound healing (**B**, x40), transwell (**C**, x200) and tube formation (**D**, x100) in antagomiR-NC group, antagomiR-150-5p group, agomiR-NC group and agomiR-150-5p group.

