# Supplementary material for: miR-150-5p suppresses tumor progression by targeting VEGFA in colorectal cancer
Source: Aging (Albany NY). 2018 Nov 26;10(11):3421–37. doi: 10.18632/aging.101656 (PMC6286841; doi:10.18632/aging.101656)
Supplement: Supplementary Figure [file aging-10-101656-s001.pdf]

SUPPLEMENTARY FIGURE

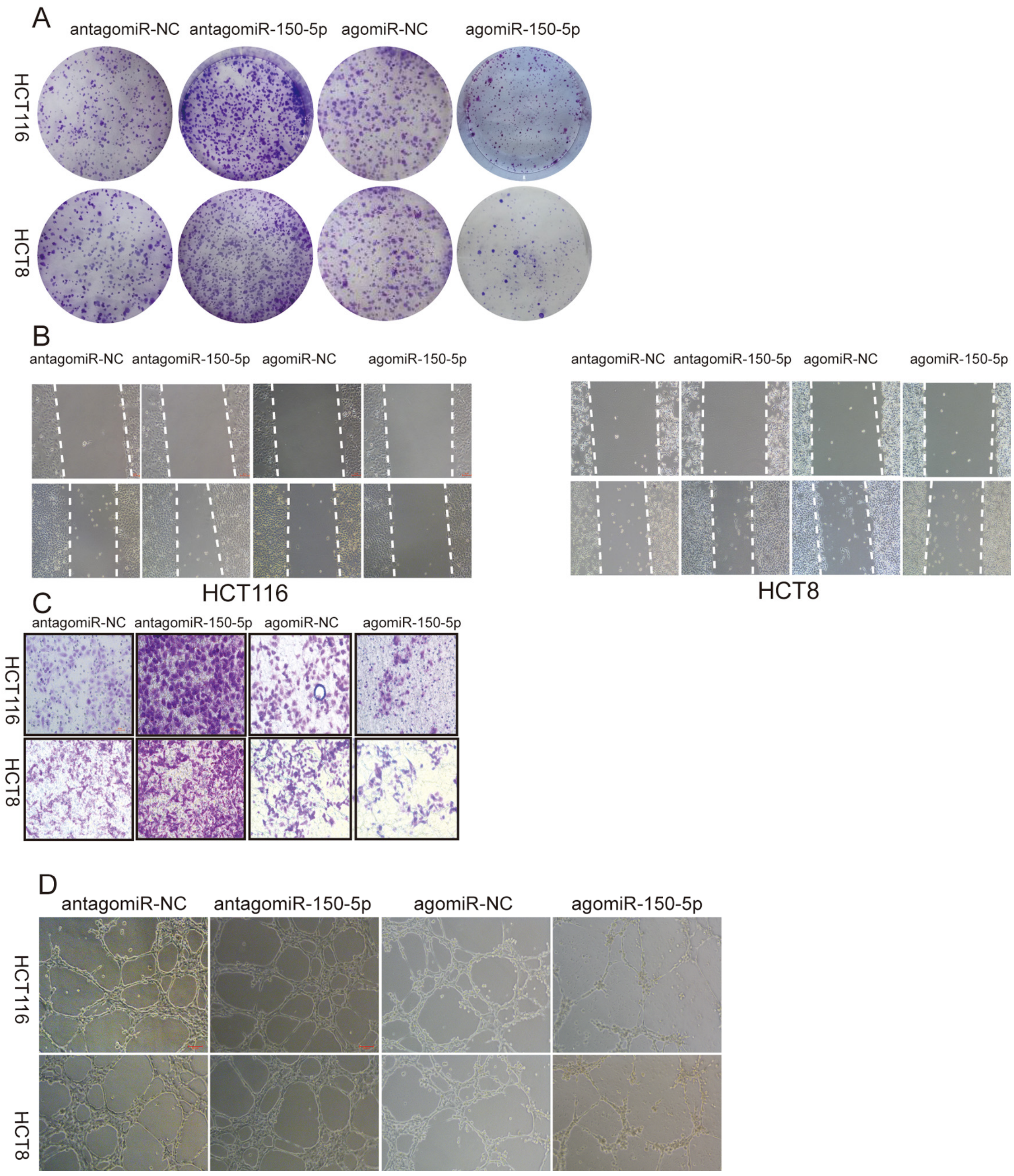

Figure S1. (**A**-**D**) Representive amages of colony formation(A), wound healing(B, ×40), transwell (**C**, ×200) and tube formation (**D**, ×100) in antagomiR-NC group, antagomiR-150-5p group, agomiR-NC group and agomiR-150-5p group.
