# Supplementary material for: miR-150-5p suppresses tumor progression by targeting VEGFA in colorectal cancer
Source: Aging (Albany NY). 2018 Nov 26;10(11):3421–37. doi: 10.18632/aging.101656 (PMC6286841; doi:10.18632/aging.101656)
Supplement: Supplementary Materials and Methods [file aging-10-101656-s002.pdf]

SUPPLEMENTARY MATERIALS AND METHODS

The sequence of scramble, siVEGFA-1, siVEGFA-2, agomiR-150-5p, agomiR-NC

scramble	5′-UUCUCCGAACGUGUCACGUTT-3′
	5′-ACGUGACACGUUCGGAGAATT-3′
siVEGFA-1	5′-CCGAAACCAUGAACUUUCUTT-3′
	5′-AGAAAGUUCAUGGUUUCGGTT-3′
siVEGFA-2	5’- GCGGCGUCGCACUGAAACUTT-3’
	5’- AGUUUCAGUGCGACGCCGCTT-3’
agomiR-150-5p	5’-UCUCCCAACCCUUGUACCAGUG-3’
	5’-CUGGUACAAGGGUUGGGAGAUU-3’
agomiR-NC	5’-UUCUCCGAACGUGUCACGUTT-3’
	5’-ACGUGACACGUUCGGAGAATT-3’
antagomiR-150-5p	5’-CACUGGUACAAGGGUUGGGAGA-3’
antagomiR-NC	5’-CAGUACUUUUGUGUAGUACAA-3’

antagomiR-150-5p and antagomiR-NC
